# Meta-regression Analysis of Sex- and Birth Year-Specific Prevalence of HBsAg and Anti-HCV Among Un-diagnosed Japanese: Data From the First-time Blood Donors, Periodical Health Checkup, and the Comprehensive Health Checkup With Lifestyle Education (Ningen Dock)

**DOI:** 10.2188/jea.JE20190055

**Published:** 2020-09-05

**Authors:** Tomoyuki Akita, Junko Tanaka, Masahiro Satake, Yingsong Lin, Takashi Wada, Kiminori Kato, Manami Inoue

**Affiliations:** 1 Department of Epidemiology, Infectious Disease Control and Prevention, Graduate School of Biomedical and Health Sciences, Hiroshima University, Hiroshima, Japan; 2 Central Blood Institute, Japanese Red Cross Society, Tokyo, Japan; 3 Department of Public Health, Aichi Medical University School of Medicine, Aichi, Japan; 4 The Large-scale Research Committee on the Usefulness of Ningen Dock Health Evaluation, Japan Society of Ningen Dock, Chiba, Japan; 5 Division of Prevention, Center for Public Health Sciences, National Cancer Center, Tokyo, Japan

**Keywords:** undiagnosed, hepatitis B, hepatitis C, large scale cohort, Japan

## Abstract

**Background:**

This study was aimed to precisely estimate the prevalence of hepatitis B surface antigen (HBsAg) and anti-hepatitis C virus antibody (anti-HCV) stratified by sex and birth year in Japan.

**Methods:**

Three large-scaled cohorts: first-time blood donors, periodic health check-up, and comprehensive health check-up with lifestyle education (Ningen dock) were used for pooled prevalence of HBsAg and anti-HCV using meta-regression.

**Results:**

Trends of birth year-specific prevalence of HBsAg and anti-HCV among the three cohorts were similar to one another, while birth year-specific pooled prevalence of HBsAg peaked in the 1941–1950 birth cohort. Prevalence of anti-HCV showed a decreasing trend by birth year.

**Conclusion:**

We could estimate the pooled prevalence of HBsAg and anti-HCV based on nationwide data. The results can be used as reference data for various countermeasures for hepatitis eradication.

## INTRODUCTION

Globally, 1.4 million people died due to hepatitis virus infection yearly.^1^^–^^3^ Mortality due to hepatitis was higher than that due to human immunodeficiency virus (HIV) or malaria, and viral hepatitis has become one of the main causes of liver cirrhosis and liver cancer.^3^ In 2015, it was estimated that 257 million people were persistently infected with hepatitis B virus (HBV) and 71 million with hepatitis C virus (HCV).^1^^,^^2^

In Japan, advanced countermeasures for hepatitis, such as prevention of vertical infection of HBV, screening of blood for transfusion, and hepatitis virus screening for residents, were first introduced ahead of others in the world.^4^ These virus infections are currently under control in Japan, with positive rates varying by birth year rather than age.

It is important to know the number of hepatitis virus carriers (persistent infection), and that number is basic information for viral hepatitis and liver cancer countermeasures. However, it is difficult to know the number of hepatitis virus carriers among the general population because some of them have no symptoms despite being infected by hepatitis virus.

The number of HBV and HCV carriers in general healthy Japanese populations has been estimated based on data from first-time blood donors and government health checkup examinees.^5^ Serological testing for hepatitis virus was performed as screening before blood donation, prenatal checkup, preoperative screening, and so on, but the type and age range of subject may vary for each screening. Blood donors generally comprise young individuals (aged 20s–30s) and tend to have relatively low prevalence of HBV and HCV. In contrast, government health checkup examinees, starting since 2002, generally target those aged 40 years and over and comprise older individuals.^5^ Meanwhile, data from populations such as those from occupational/work sites are lacking.^5^ Therefore, aggregating estimates by adding large-scale data from occupational/worksite populations will increase the representativeness of HBV and HCV carrier rates in Japan. Furthermore, it is crucial to estimate positive rates by birth year rather than age because HBV or HCV infection is more likely to have been influenced by cohort effect than by age effect. It is reported that the incidence rates of HBV and HCV infection have been very low after 2000,^6^^,^^7^ and the sanitation status during childhood and the preventive measures (such as national program for passive and active immunoprophylaxis of babies born to carrier mothers and HBV/HCV screening of blood transfusion^4^^,^^8^^,^^9^) affect the chronic infection with HBV. Therefore, in this study, we stratified estimated prevalence rates by birth year, not by age.

In this study, we estimated the prevalence of HBV and HCV among the general population in Japan with high precision using meta-regression based on results of studies among first-time blood donors, periodic health checkup recipients, and the examinees of the comprehensive health checkup with lifestyle education (Ningen dock).

## METHOD

### Study subjects

The following three large-scaled cohorts were used to estimate the prevalence of hepatitis B surface antigen (HBsAg) and antibody to hepatitis C virus (anti-HCV) in Japan.

### First-time blood donors

During 1995 to 2000, 3,485,648 peoples (aged 16–69 years) donated whole blood or apheresis products as their first time. Their sera were tested routinely for serum markers of HBV and HCV infections.

HBsAg was determined using reversed-passive hemagglutination with reagents made in-house by the Japanese Red Cross Blood Center. Anti-HCV was determined using passive hemagglutination with commercial kits (the second generation HCV PHA [Dinabott, Tokyo, Japan] and HCV PA [Ortho Diagnostics, Tokyo, Japan]). The cutoff level for the hemagglutination test of HBsAg was 22 and that of anti-HCV was either 25 or 24.

### Examinees of hepatitis virus infections through periodic health check-ups

Since 2002 in Japan, individuals who turned 40, 45, 50, 55, 60, 65 and 70 years were offered tests for hepatitis viruses through a 5-year national project on periodic health check-ups. Through the end of the project in 2006, 6,228,967 individuals were tested for HBV and 6,204,968 individuals had received tests for HCV. However, sex-specific data among them was not published, so this data was used only for estimation of prevalence in both sexes.

As for HCV screening, each subject was screened using the flowchart of HCV screening from the Ministry of Health, Labour and Welfare Japan (MHLW), and the result of screening was published as “high possibility of HCV infection” not “anti-HCV positive”. In this study, we assumed that 70% of anti-HCV positive person are persistently infected with HCV; therefore, we transformed the positive rate of “high possibility of HCV infection” by 1.00/0.70 multiplied.

### Examinees of hepatitis virus infections through the comprehensive health checkup with lifestyle education (Ningen dock)

Information on HBV and HCV infection among the Ningen Dock examinees in 2014 was extracted from the Japan Ningen Dock Study database after approval by the Research Ethics Committee of the Japan Society of Ningen Dock (approval number 0010, October 2017). The data comprised summary tables with the number of positive and negative infections by sex and birth year at the time of the checkup, without any personal identifiers. HBV infection was determined at the checkup via the detection of hepatitis B surface antigen (HBsAg), and HCV via the presence of anti-HCV antibody in serum (anti-HCV). The data included the results for the 1.6 million individuals conducted. Data of 1,153,261 examinees of HBV and 742,783 examinees of HCV were used.

### Statistical analysis

Meta-regression analysis was performed to estimate birth year-specific pooled prevalence of HBsAg and anti-HCV by three categories (both sexes, male, and female), using aggregated data of two or three cohorts. We assumed that relation between prevalence of HBsAg or anti-HCV and birth year were approximated by a cubic spline regression model:

$$f(x)=\sum_{j=1}^{q}\alpha_{j}B_{j}(x)$$

where *B_j_*(*x*) is basis function of local cubic polynomials

$$\begin{array}{r} B_{j}(x)=a_{j}+b_{j}(x-x_{j})+c_{j}(x-x_{j}{)}^{2}+d_{j}(x-x_{j}{)}^{3}\\ \;(x_{j}\leq x<x_{j}+1) \end{array}$$

and *x* is dummy code of birth year such as *x* = 1 for 1931–35; *x* = 2 for 1936–40, …; *x* = 11 for 1981–85, *q* = 11 is number of birth-cohorts.^10^ The cubic spline method uses a set of third-degree polynomials spliced together such that the resulting curve is continuous and smooth at the splices (knot points). Fitting a cubic spline curve was estimated based on weight of each cohort’s sample size and penalty least-squared method, which means that the estimation is done by minimizing an objective function that is a combination of the sum of squares error and a penalty for curvature integrated over the curve extent given by Reinsch.^11^ We also calculated conservative 95% confidence intervals (CIs) of pooled prevalence using Wald’s CI with use of theoretical maximum value of variance of pooled prevalence.

All statistical analyses were performed using JMP 13 (SAS Institute Inc., Cary, NC, USA).

### Ethical considerations

This study used data of the first-time blood donors and examinees of hepatitis virus infections through periodic health check-ups from the already published papers available as secondary data in the public domain; ethics approval was not required for them. Information on HBV and HCV infection among the Ningen Dock examinees in 2014 was extracted from the Japan Ningen Dock Study database after approval by the Research Ethics Committee of the Japan Society of Ningen Dock (approval number 0010, October 2017).

## RESULTS

**Figure 1.**
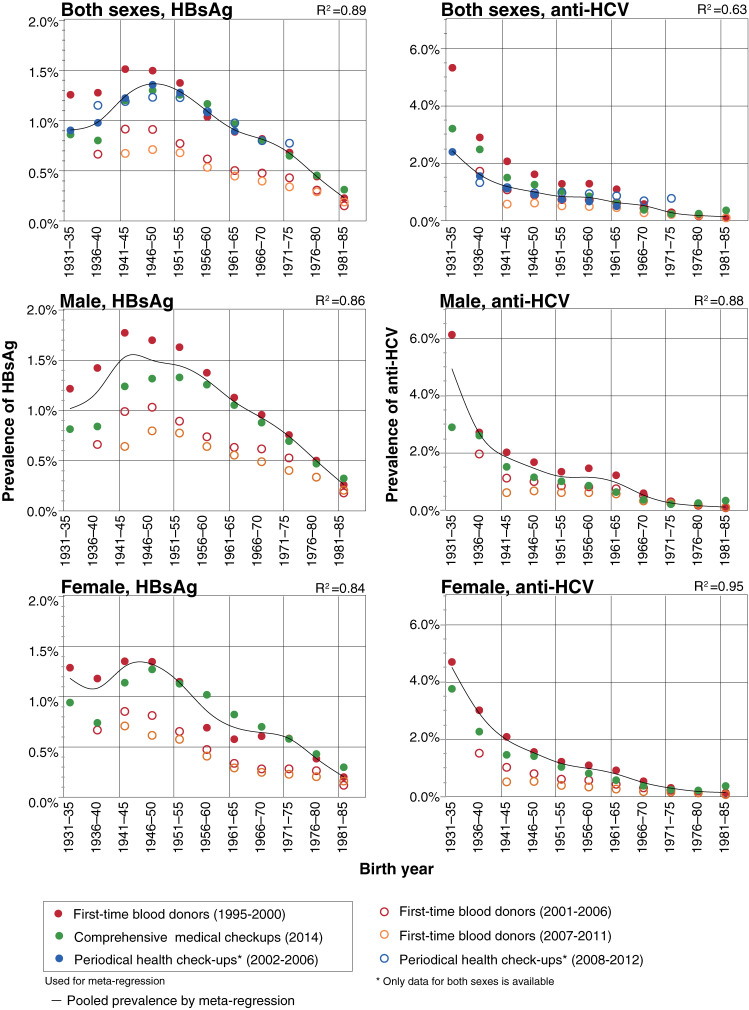
Pooled prevalence of HBsAg and anti-HCV by meta-regression model. This figure showed the HBsAg and anti-HCV prevalence of each cohort study and the pooled prevalence of HBsAg and anti-HCV using meta-regression model. The three left sided figures represent for HBsAg prevalence while the three right sided ones represent for anti-HCV prevalence. The two figures in upper row represent for the overall prevalence while the middle and lower rows indicate for the respective prevalence in male and female respectively. In each figure, the earlier cohort studies were showed with red, green and blue dots while the later studies were indicated with red, orange and blue colored rings. The line in every figure indicates for the resultant pooled prevalence by meta-regression model.

Sex- and birth year-specific prevalence of HBsAg and anti-HCV among the three cohorts are shown in Table 1 and Figure 1 as dots. For reference, two cohorts of first-time blood donors (2001–2006 and 2007–2011) and periodic health check-ups (2008–2012) are also shown as white circles.

**Table 1.** Prevalence of HBsAg and anti-HCV among three large cohorts and pooled prevalence using meta-regression

Gender	Birth year	Hepatitis B surface antigen (HBsAg)							Anti-hepatitis C virus antibody (anti-HCV)						
	
		FT-BD		CMC		PHC		Pooled prevalence	FT-BD		CMC		PHC		Pooled prevalence
					
		*N*	Prevalence	*N*	Prevalence	*N*	Prevalence		*N*	Prevalence	*N*	Prevalence	*N*	Prevalence^a^	
**Both sex**	1991–1995				1,063	0.19%						901	0.55%			
	1986–1990			5,854	0.24%						4,495	0.40%			
	1981–1985	582,415	0.23%	23,367	0.31%			**0.23% (0.16–0.30)**	582,415	0.13%	16,369	0.35%			**0.13% (0.04–0.22)**
	1976–1980	1,294,064	0.44%	93,131	0.45%			**0.44% (0.40–0.48)**	1,294,064	0.17%	62,102	0.24%			**0.17% (0.13–0.21)**
	1971–1975	635,083	0.68%	181,504	0.65%			**0.67% (0.63–0.71)**	635,083	0.28%	117,516	0.22%			**0.28% (0.25–0.31)**
	1966–1970	294,606	0.82%	186,610	0.81%			**0.81% (0.76–0.86)**	294,606	0.57%	120,393	0.37%			**0.52% (0.48–0.56)**
	1961–1965	177,841	0.89%	188,929	0.97%	613,960	0.89%	**0.91% (0.84–0.98)**	177,841	1.09%	122,909	0.61%	611,146	0.50%	**0.63% (0.56–0.70)**
	1956–1960	131,974	1.04%	176,784	1.17%	497,589	1.08%	**1.09% (1.01–1.17)**	131,974	1.28%	114,554	0.84%	495,032	0.66%	**0.80% (0.72–0.88)**
	1951–1955	115,046	1.38%	136,644	1.26%	679,893	1.28%	**1.29% (1.20–1.38)**	115,046	1.28%	87,346	1.02%	675,350	0.74%	**0.84% (0.74–0.94)**
	1946–1950	117,017	1.50%	87,993	1.30%	950,508	1.36%	**1.37% (1.27–1.47)**	117,017	1.61%	53,751	1.25%	947,438	0.90%	**0.99% (0.87–1.11)**
	1941–1945	81,460	1.51%	42,052	1.20%	1,085,119	1.22%	**1.24% (1.10–1.38)**	81,460	2.06%	25,146	1.50%	1,081,854	1.11%	**1.19% (1.01–1.37)**
	1936–1940	44,927	1.28%	19,700	0.80%	1,268,304	0.98%	**0.99% (0.83–1.15)**	44,927	2.90%	11,583	2.48%	1,264,496	1.55%	**1.60% (1.28–1.92)**
	1931–1935	11,215	1.26%	7,550	0.86%	1,057,469	0.90%	**0.91% (0.62–1.20)**	11,215	5.32%	4,434	3.20%	1,054,472	2.39%	**2.43% (1.77–3.09)**
	1926–1930			1,972	0.35%						1,224	2.78%			
	1921–1925			108	0.00%						60	3.33%			

**Male**	1991–1995			510	0.39%						390	0.26%			
	1986–1990			3,098	0.29%						2,367	0.21%			
	1981–1985	273,842	0.26%	13,304	0.32%			**0.26% (0.16–0.36)**	273,842	0.11%	9,395	0.34%			**0.12% (0.00–0.24)**
	1976–1980	644,720	0.50%	55,931	0.47%			**0.50% (0.44–0.56)**	644,720	0.16%	37,322	0.25%			**0.16% (0.11–0.21)**
	1971–1975	360,266	0.76%	108,549	0.69%			**0.74% (0.68–0.80)**	360,266	0.27%	70,061	0.21%			**0.26% (0.22–0.30)**
	1966–1970	177,553	0.96%	111,845	0.88%			**0.93% (0.86–1.00)**	177,553	0.60%	72,178	0.36%			**0.53% (0.47–0.59)**
	1961–1965	100,074	1.13%	116,671	1.05%			**1.09% (1.00–1.18)**	100,074	1.22%	76,441	0.64%			**0.97% (0.88–1.06)**
	1956–1960	66,512	1.38%	109,317	1.26%			**1.30% (1.19–1.41)**	66,512	1.47%	71,695	0.86%			**1.15% (1.04–1.26)**
	1951–1955	54,064	1.63%	86,463	1.33%			**1.44% (1.31–1.57)**	54,064	1.35%	56,120	1.01%			**1.18% (1.05–1.31)**
	1946–1950	49,484	1.70%	54,516	1.32%			**1.50% (1.35–1.65)**	49,484	1.68%	33,581	1.15%			**1.47% (1.31–1.63)**
	1941–1945	30,852	1.77%	25,652	1.24%			**1.53% (1.33–1.73)**	30,852	2.02%	15,552	1.52%			**1.85% (1.60–2.10)**
	1936–1940	17,849	1.42%	12,138	0.84%			**1.19% (0.95–1.43)**	17,849	2.72%	7,129	2.61%			**2.69% (2.25–3.13)**
	1931–1935	4,933	1.22%	4,791	0.81%			**1.02% (0.62–1.42)**	4,933	6.12%	2,865	2.90%			**4.94% (4.03–5.85)**
	1926–1930			1,310	0.46%						848	2.71%			
	1921–1925			73	0.00%						41	2.44%			

**Female**	1991–1995			553	0.00%						511	0.78%			
	1986–1990			2,756	0.18%						2,128	0.61%			
	1981–1985	308,573	0.20%	10,063	0.30%			**0.20% (0.09–0.31)**	308,573	0.14%	6,974	0.37%			**0.15% (0.01–0.29)**
	1976–1980	649,344	0.38%	37,200	0.43%			**0.39% (0.32–0.46)**	649,344	0.18%	24,780	0.22%			**0.18% (0.12–0.24)**
	1971–1975	274,817	0.58%	72,955	0.58%			**0.58% (0.52–0.64)**	274,817	0.31%	47,455	0.24%			**0.30% (0.25–0.35)**
	1966–1970	117,053	0.61%	74,765	0.70%			**0.64% (0.57–0.71)**	117,053	0.54%	48,215	0.38%			**0.49% (0.42–0.56)**
	1961–1965	77,767	0.58%	72,258	0.82%			**0.70% (0.62–0.78)**	77,767	0.92%	46,468	0.58%			**0.79% (0.69–0.89)**
	1956–1960	65,462	0.69%	67,467	1.02%			**0.86% (0.76–0.96)**	65,462	1.10%	42,859	0.81%			**0.98% (0.86–1.10)**
	1951–1955	60,982	1.15%	50,181	1.13%			**1.14% (1.01–1.27)**	60,982	1.22%	31,226	1.04%			**1.16% (1.02–1.30)**
	1946–1950	67,533	1.35%	33,477	1.27%			**1.32% (1.17–1.47)**	67,533	1.56%	20,170	1.41%			**1.53% (1.34–1.72)**
	1941–1945	50,608	1.35%	16,400	1.14%			**1.30% (1.11–1.49)**	50,608	2.09%	9,594	1.46%			**1.99% (1.72–2.26)**
	1936–1940	27,078	1.18%	7,562	0.74%			**1.09% (0.86–1.32)**	27,078	3.01%	4,454	2.27%			**2.91% (2.43–3.39)**
	1931–1935	6,282	1.29%	2,759	0.94%			**1.18% (0.72–1.66)**	6,282	4.70%	1,569	3.76%			**4.51% (3.43–5.59)**
	1926–1930			662	0.15%						376	2.93%			
	1921–1925			35	0.00%						19	5.26%			

CMC, Comprehensive Health checkups (Ningen dock) (2014); FT-BD, First-time blood donors (1995–2000); PHC, Periodical health check-ups (2002–2006).

^a^70% of anti-HCV positives were assumed as carriers.

As for HBsAg, prevalence of three cohorts are close to one another and spline curve is well fitted except for the people whose birth year is earlier than around 1950. As for the people whose birth year is earlier than around 1950, prevalence of HBsAg among first-time blood donors is higher than that among Ningen Dock examinees. Regression curve of HBsAg based on cubic spline curve shows peaks in 1941–45 and 1946–50 birth cohorts.

As for anti-HCV, prevalence among the three cohorts were close to one another in people whose birth year is later than around 1940. For the people whose birth year is earlier than 1940, prevalence of anti-HCV among periodic health checkup attendees is lower than that among Ningen Dock examinees and first-time blood donors. The regression curve of anti-HCV shows a decreasing trend by birth year.

## DISCUSSION

First-time blood donors is a large cohort; however, most of them are in a young generation who were aged 39 years old or younger, and middle and elder generation members are not common in this cohort. On the other hand, many of the examinees of periodic health check-ups or Ningen dock were 40 years or older. In this study, we combined some prevalence rates among large-scaled populations and estimated the precise prevalence as a pooled one. Birth year-specific pooled prevalence of HBsAg peaked in the 1941–1950 birth cohort. That of anti-HCV shows a decreasing trend by birth year.

In Japan, large-scale sero-prevalence studies among all first-time blood donors were conducted three times: 1995–2000, 2001–2006, and 2007–2011.^5^^,^^12^^,^^13^ During each period of study, screening of hepatitis B and C was promoted, and residents who were already known to be infected never go for blood donation and are not involved in prevalence study. For this reason, the prevalence of both diseases seems to be underestimated. Therefore, we used prevalence in 1995–2000, which was similar to that of the Ningen dock examinees among cohorts who were born in later years, for meta-regression. Although, for the prevalence among cohorts who were born in earlier years are different, *R*^2^ ranged from 0.84–0.95. It is suggested that members of two big cohorts who were born in later years were very similar.

In fact, hepatitis virus carriers can be categorized into four groups: a) undiagnosed carriers, b) patients, c) carriers who know their own infection status but have not been seen at hospital, and d) new infection.^13^ In this study, we estimated the prevalence of undiagnosed carriers among the general population.

Age and birth cohort effects can be seen in liver cancer mortality, especially in the cohort who were born in the 1930s.^14^ In Japan, the main cause of liver cancer is persistent HBV and HCV infection. In this study, we observed a peak of prevalence of anti-HCV among the 1931–1935 birth cohort.

Low prevalence of HBsAg among the young generation was observed in this study. According to mathematical model study of infectious routes of HBV infection, the number of HBV carriers with horizontal infection was estimated to be 2.17–3.93 times higher than that with vertical infection among 1950–1959 birth cohorts, while the ratio was decreased to 0.06–0.37 times among the 1980–1985 birth cohorts.^15^ Countermeasures for preventing mother-to-infant infection of HBV were introduced since 1986, and prevalence of HBsAg is expected to become very low.

The estimated prevalence of hepatitis virus in this study can also be used for analysis of correlation between hepatitis virus carrier and liver cancer, as well as risk factor analysis of liver cancer. It is also important for updating the data for monitoring of hepatitis virus infection transmission.

In conclusion, we estimated the pooled prevalence of HBsAg and anti-HCV based on nationwide data. The results will be important reference data for various countermeasures for hepatitis eradication.
